# Sociodemographic and Psychological Correlates of Compliance with the COVID-19 Public Health Measures in France

**DOI:** 10.1017/S0008423920000335

**Published:** 2020-04-23

**Authors:** Sylvain Brouard, Pavlos Vasilopoulos, Michael Becher

**Affiliations:** 1 CEVIPOF, Sciences Po, 98 rue de l'Université, 75007, Paris, France; 2 Department of Politics, University of York, Heslington Lane, York, YO10 5DD, United Kingdom; 3 Institute for Advanced Study in Toulouse, Université Toulouse 1 Capitole, 1 esplanade de l'Université, 31080, Toulouse, France

## Abstract

The COVID-19 disease was first identified in Wuhan, China, in December 2019, having since spread rapidly across the world. The infection and mortality rates of the disease have forced governments to implement a wave of public health measures. Depending on the context, these range from the implementation of simple hygienic rules to measures such as social distancing or lockdowns that cause major disruptions in citizens’ daily lives. The success of these crucial public health measures rests on the public's willingness to comply. However, individual differences in following the official public health recommendations for stopping the spread of COVID-19 have not yet to our knowledge been assessed. This study aims to fill this gap by assessing the sociodemographic and psychological correlates of implementing public health recommendations that aim to halt the COVID-19 pandemic. We investigate these associations in the context of France, one of the countries that has been most severely affected by the pandemic, and which ended up under a nationwide lockdown on March 17. In the next sections we describe our theoretical expectations over the associations between sociodemographics, personality, ideology, and emotions with abiding by the COVID-19 public health measures. We then test these hypotheses using data from the French Election Study.

## Introduction

The COVID-19 disease was first identified in Wuhan, China, in December 2019, having since spread rapidly across the world. The infection and mortality rates of the disease have forced governments to implement a wave of public health measures. Depending on the context, these range from the implementation of simple hygienic rules to measures such as social distancing or lockdowns that cause major disruptions in citizens’ daily lives. The success of these crucial public health measures rests on the public's willingness to comply. However, individual differences in following the official public health recommendations for stopping the spread of COVID-19 have not yet to our knowledge been assessed. This study aims to fill this gap by assessing the sociodemographic and psychological correlates of implementing public health recommendations that aim to halt the COVID-19 pandemic. We investigate these associations in the context of France, one of the countries that has been most severely affected by the pandemic, and which ended up under a nationwide lockdown on March 17. In the next sections we describe our theoretical expectations over the associations between sociodemographics, personality, ideology, and emotions with abiding by the COVID-19 public health measures. We then test these hypotheses using data from the French Election Study.

## Theoretical Expectations

### Age

The key demographic that we expect to be associated with adherence to the public health recommendations is age. Given that older individuals are at greater risk of dying from the disease, self-interest suggests that age will be positively associated with personally implementing measures to stop the spread of the virus. At the same time, some of the health measures reduce social interactions, which are more frequent among the young. Consequently, we anticipate that age will be positively associated with complying with the COVID-19 measures (H_1_).

### Personality Traits

We are also interested in considering the role of personality in public compliance with the measures. To this end we employ the Big Five personality framework (McCrae and John, 1992). The model argues that individual differences in personality can be summarized in five dimensions, namely *openness to experience*, *conscientiousness*, *extraversion*, *agreeableness*, and *neuroticism*. We believe that at least three of the five personality dimensions should be associated with compliance: conscientiousness, extraversion, and neuroticism. Conscientious individuals tend to be duty-bound and have a high sense of obligation. Therefore, we anticipate that individuals scoring high in conscientiousness will be more likely to abide with the public health measures (H_2_). On the other hand, we expect that extraversion should be negatively associated with implementing public health recommendations (H_3_). Extroverted individuals should find it harder compared to introverted individuals to comply with isolating measures that disrupt sociability. Finally, we hypothesize that neuroticism should be positively associated with compliance (H_4_). This is because individuals scoring high in neuroticism tend to be more sensitive to threat and more risk-averse compared to the emotionally stable.

### Ideology

In addition to differences in personality, we anticipate that political ideology should be associated with the propensity to implement changes in daily life as a result of the COVID-19 pandemic. Research shows that individuals who place themselves in ideological extremes are both more distrusting of the state and its powers as well as more prone to endorsing conspiracy theories (Van Prooijen et al., 2015). We therefore anticipate that ideological extremity will be negatively associated with abiding by the COVID-19 public health measures (H_5_).

### Fear

Our final hypothesis has to do with fear. A large stream of research in social and political psychology has shown that experiencing fear toward a threatening stimulus is associated with behavioural and attitudinal change (Marcus et al., 2000). Moreover, fear and anxiety render individuals more likely to opt for risk-aversive behavioural strategies (Lerner and Keltner, 2001) and to show increased compliance with authority (Vasilopoulos et al., 2018). These findings lead us to hypothesize that experiencing fear in the light of the COVID-19 pandemic should be positively associated with complying with the public health measures (H_6_).

## Data and Methods

Data come from the French National Election Study, a panel survey begun in November 2015. The sample was constructed with the use of quota controls for age, gender, and occupation, and stratified by size of community and region of residence (Ile de France, North-West, North-East, South-West, South-East). On March 16–17, 2020, we added an additional wave to the panel through a random selection of 1,010 from the panel's 24,369 respondents, with questions focusing on the COVID-19 disease.

The dependent variable is an index of compliance with public health measures aimed at slowing the spread of the COVID-19 disease. It measures the extent to which French citizens followed the voluntary recommendations issued by the French government. We asked respondents whether they have changed daily behaviours as a result of the coronavirus epidemic on a scale ranging from 0 (not at all) to 10 (very much). We presented to them a range of different behaviours recommended by the French public health authorities. These included “Washing your hands more often and/or longer”; “Coughing or sneezing into your elbow or a handkerchief”; “Stopping greeting by shaking hands or kissing”; “Keeping a distance of one meter from other people outside your home”; “Having reduced your trips”; “Avoiding crowded places”; and “Having stopped meeting your friends.” The scale had high reliability (α = 0.89), and a factor analysis showed that all items loaded on one factor. Table A1 in the Appendix (see Supplementary Materials) presents the mean and standard deviation for the continuous and dummy variables.

As for the independent variables, our models include age as well as controls for gender, level of education, and size of community. Regarding the psychological characteristics, we include individual differences in the Big Five personality traits using the Ten Item Personality Inventory (TIPI; Gosling et al. 2003) administered in September 2016 (Wave 6). The prior measurement of the personality items mitigates concern about priming and reverse causality. Furthermore, we included a measure of ideological self-placement, using an item that asks respondents to place themselves on a scale that ranges from 0 (“Left”) to 10 (“Right”). As we expect the association of ideology with compliance to be nonlinear, we also included the squared term of ideology. Moreover, we have included two items measuring trust toward the President and scientists. These have been measured using a four-point scale that ranges from 1 (not trust at all) to 4 (trust very much) which were subsequently recoded into dummy variables indicating trust and non-trust.

Finally, our list of independent variables includes emotional reactions. Following the research design used to assess emotional responses to the 2015 Paris terror attacks (Vasilopoulos et al., 2019), we asked respondents the extent to which they experience fear, anger, and hope “when thinking about the situation with the COVID-19 in France” on a scale ranging from 0 (not at all) to 10 (extremely). While our hypothesis only involves the role of fear, past research has found that these three emotional dimensions of fear, anger, and hope are intercorrelated, and hence it is recommended to control for all, even when one is interested in assessing the effect of only one (Vasilopoulos et al., 2019).

## Results

We estimated four linear regression models adding covariates incrementally, given that personality and demographic characteristics are considered causally prior to ideology, trust, and emotional reactions (Carney et al., 2008).^1^ Model 1 includes only demographic characteristics. Model 2 adds the Big Five personality dimensions. Model 3 includes ideology and trust toward the government and scientists, and, finally, Model 4 adds emotions. The findings are presented in Table 1. In order to facilitate the interpretation of the results, all variables have been recoded from 0 to 1.

**Table 1:** Demographic and Attitudinal Correlates of Compliance

	Model 1	Model 2	Model 3	Model 4
Age	0.18***	0.13***	0.16***	0.13**
	(0.04)	(0.04)	(0.04)	(0.04)
Female	0.09***	0.08***	0.08***	0.07***
	(0.01)	(0.01)	(0.01)	(0.01)
Middle education	–0.01	–0.02	–0.03	–0.03
	(0.02)	(0.02)	(0.02)	(0.02)
High education	0.03	0.01	0.00	0.00
	(0.02)	(0.02)	(0.02)	(0.02)
*Size of community (ref. < 5000 inhabitants)*				
2,000–9,999	–0.02	–0.02	–0.02	–0.01
	(0.02)	(0.02)	(0.02)	(0.02)
10,000–49,999	–0.00	–0.00	–0.00	–0.00
	(0.02)	(0.02)	(0.02)	(0.02)
50,000–199,999	–0.04^#^	–0.04^#^	–0.05^#^	–0.03
	(0.02)	(0.02)	(0.02)	(0.02)
200,000 or more	–0.02	–0.02	–0.03	–0.03^#^
	(0.02)	(0.02)	(0.02)	(0.02)
*Personality*				
Openness to new experiences		–0.02	–0.02	–0.02
		(0.03)	(0.03)	(0.03)
Conscientiousness		0.10*	0.10*	0.09*
		(0.04)	(0.05)	(0.04)
Extraversion		–0.07*	–0.07*	–0.07*
		(0.03)	(0.03)	(0.03)
Agreeableness		0.03	0.05	0.05
		(0.05)	(0.05)	(0.05)
Neuroticism		–0.03	–0.02	–0.08*
		(0.03)	(0.04)	(0.03)
Ideology			0.24*	0.21^#^
			(0.12)	(0.11)
Ideology squared			–0.24*	–0.24*
			(0.11)	(0.10)
Trust in scientists			0.04	0.04
			(0.03)	(0.02)
Trust in the President			0.02	0.02^#^
			(0.01)	(0.01)
Emotions				
Fear				0.23***
				(0.03)
Hope				0.01
				(0.03)
Anger				0.01
				(0.02)
Constant	0.71***	0.70***	0.59***	0.51***
	(0.03)	(0.06)	(0.08)	(0.07)
				
Observations	870	794	690	690
*R*^2^	0.091	0.105	0.146	0.261

Standard errors in parentheses.

^#^ *p* < 0.1, * *p* < 0.05, ** *p* < 0.01, *** *p* < 0.001.

Starting with Model 1, the results suggest that age is positively associated with complying with the measures. Further, women are more likely to have changed their behaviour compared to men. However, we find that education is not associated with public compliance. Further, our findings indicate that conscientiousness is positively associated with having changed behaviour in line with the recommendations. This is in line with H_2_. Moreover, and confirming our expectations (H_3_), extraversion is negatively associated with having changed one's daily behaviours in the light of the pandemic. Finally and unexpectedly, neuroticism has a negative association when emotions are taken into account (Model 4). This finding could be attributed to the tendency of neurotic individuals to appraise that their capacity for coping with a threatening stimulus is low, which may lead to inaction (Penley and Tomaka, 2002).

Model 3 adds the association of trust, ideology, and ideology squared with compliance to public health instructions. Results illustrate that, in line with H_5_, ideological extremity is associated with a reduced adherence to public health recommendations. At the same time, compliance increases as one moves from the left to the right end of the ideology scale. Finally, results from Model 4 provide strong empirical confirmation for H_6_ over the association between fear and compliance.

## Discussion

Facing their largest health crisis in decades due the COVID-19 pandemic, governments adopted health measures at an unprecedented scale to slow the spread of the virus. However, enforcement is costly, and compliance by citizens cannot be taken for granted. Drawing on individual panel data, we find that some basic sociodemographic characteristic as well as personality traits are relevant predictors of compliance with these measures in France. A potential limitation of the results is that possible social desirability tendencies by a part of the respondents may overstate the true extent of compliance. In addition, compliance may have increased in the weeks after the survey was administered given the increase in COVID-19 cases. Overall, our results provide insight into the individual foundations of compliance in the times of COVID-19 that can provide the basis for policy makers to evaluate the effectiveness of their measures as well as for future research on the topic.

## Note

1 As a robustness test, we further controlled for department fixed effects in order to capture unobserved heterogeneity across geographical areas. This also accounts for differences in the spread of the COVID-19 and differences in the enforcement ability of the measures between departments. Results are presented in Table A2 in the online Appendix.

## Supplementary material

For supplementary material accompanying this paper visit http://dx.doi.org/10.1017/S0008423920000335. click here to view supplementary material
