# Supplementary material for: Sociodemographic and Psychological Correlates of Compliance with the COVID-19 Public Health Measures in France
Source: Can J Polit Sci. 2020 Apr 23:1–6. doi: 10.1017/S0008423920000335 (PMC7203163; doi:10.1017/S0008423920000335)
Supplement: Supplementary file 1 [file S0008423920000335sup001.docx]

**COVID CJPS ONLINE APPENDIX**

**Table A1: Summary statistics for continuous and dummy variables**

| Variable | Mean | Std. Dev. | Min | Max |
| --- | --- | --- | --- | --- |
|  |  |  |  |  |
| Compliance (dependent variable) | 0.83 | 0.19 | 0.00 | 1.00 |
| Age | 0.37 | 0.20 | 0.00 | 1.00 |
| Openness to New Experiences | 0.59 | 0.18 | 0.00 | 1.00 |
| Conscientiousness | 0.78 | 0.17 | 0.00 | 1.00 |
| Extraversion | 0.43 | 0.19 | 0.00 | 1.00 |
| Agreeableness | 0.69 | 0.17 | 0.00 | 1.00 |
| Neuroticism | 0.39 | 0.20 | 0.00 | 1.00 |
| Trust in scientists | 0.86 | 0.35 | 0.00 | 1.00 |
| Trust in the President | 0.45 | 0.50 | 0.00 | 1.00 |
| Fear | 0.59 | 0.26 | 0.00 | 1.00 |
| Hope | 0.55 | 0.25 | 0.00 | 1.00 |
| Anger | 0.52 | 0.32 | 0.00 | 1.00 |

**Table A2. Demographic and Attitudinal Correlates of Compliance with Public Health Instructions: Accounting for Department Fixed-Effects**

|  |  |  |  |  |
| --- | --- | --- | --- | --- |
| Age | 0.19*** | 0.15*** | 0.17*** | 0.15*** |
|  | (0.03) | (0.03) | (0.04) | (0.04) |
| Female | 0.09*** | 0.09*** | 0.09*** | 0.07*** |
|  | (0.01) | (0.02) | (0.02) | (0.01) |
| Middle education | -0.01 | -0.01 | -0.02 | -0.03 |
|  | (0.02) | (0.02) | (0.02) | (0.02) |
| High education | 0.03 | 0.02 | 0.00 | 0.00 |
|  | (0.02) | (0.02) | (0.02) | (0.02) |
| *Size of community (ref. < 2,000 inhabitants)* | | | | |
| 2,000-9,999 | -0.03 | -0.04^#^ | -0.04 | -0.03 |
|  | (0.02) | (0.02) | (0.03) | (0.02) |
| 10,000-49,999 | -0.01 | -0.01 | -0.01 | -0.01 |
|  | (0.02) | (0.02) | (0.02) | (0.02) |
| 50,000-199,999 | -0.05^#^ | -0.05^#^ | -0.06^#^ | -0.05^#^ |
|  | (0.03) | (0.03) | (0.03) | (0.03) |
| 200,000 or more | -0.04^#^ | -0.04^#^ | -0.04^#^ | -0.05* |
|  | (0.03) | (0.02) | (0.02) | (0.02) |
| *Personality* |  |  |  |  |
| Openness to New Experiences |  | -0.04 | -0.03 | -0.03 |
|  |  | (0.03) | (0.04) | (0.03) |
| Conscientiousness |  | 0.11* | 0.12* | 0.11** |
|  |  | (0.04) | (0.05) | (0.04) |
| Extraversion |  | -0.06 | -0.07 | -0.07^#^ |
|  |  | (0.04) | (0.04) | (0.04) |
| Agreeableness |  | 0.02 | 0.03 | 0.04 |
|  |  | (0.05) | (0.05) | (0.04) |
| Neuroticism |  | -0.03 | -0.02 | -0.08** |
|  |  | (0.03) | (0.03) | (0.03) |
| Ideology |  |  | 0.23^#^ | 0.16 |
|  |  |  | (0.13) | (0.12) |
| Ideology squared |  |  | -0.23^#^ | -0.21^#^ |
|  |  |  | (0.12) | (0.11) |
| Trust in scientists |  |  | 0.03 | 0.03 |
|  |  |  | (0.03) | (0.03) |
| Trust in president |  |  | 0.02 | 0.03^#^ |
|  |  |  | (0.02) | (0.02) |
| Fear |  |  |  | 0.24*** |
|  |  |  |  | (0.03) |
| Hope |  |  |  | -0.02 |
|  |  |  |  | (0.03) |
| Anger |  |  |  | 0.02 |
|  |  |  |  | (0.02) |
| Constant | 0.71*** | 0.71*** | 0.61*** | 0.53*** |
|  | (0.02) | (0.06) | (0.09) | (0.09) |
|  |  |  |  |  |
| Observations | 870 | 794 | 690 | 690 |
| R-squared | 0.09 | 0.11 | 0.15 | 0.26 |
| Number of ALOC1 | 92 | 92 | 92 | 92 |

Standard errors in parentheses are clustered by department. All models include department fixed effects

(*R^2^* is net of fixed effects).

^#^ *p* < 0.1, ^*^ *p* < 0.05, ^**^ *p* < 0.01, ^***^ *p* < 0.001
